# Essential Trace Elements in Three Species of Dolphins Stranded in the Croatian Part of the Adriatic Sea from 1995 to 2013

**DOI:** 10.3390/ani15111535

**Published:** 2025-05-23

**Authors:** Maja Đokić, Nina Bilandžić, Marija Sedak, Tomislav Bolanča, Tomislav Gomerčić, Martina Đuras, Miroslav Benić

**Affiliations:** 1 Laboratory for Residue Control, Department of Veterinary Public Health, Croatian Veterinary Institute, Savska Cesta 143, 10000 Zagreb, Croatia; bilandzic@veinst.hr (N.B.); sedak@veinst.hr (M.S.); 2 Department of Analytical Chemistry, Faculty of Chemical Engineering and Technology, University of Zagreb, Trg Marka Marulića 19, 10000 Zagreb, Croatia; tbolanca@fkit.unizg.hr; 3 Department of Biology, Faculty of Veterinary Medicine, University of Zagreb, Heinzelova 55, 10000 Zagreb, Croatia; tomislav.gomercic@vef.unizg.hr; 4 Department of Anatomy, Histology and Embryology, Faculty of Veterinary Medicine, University of Zagreb, Heinzelova 55, 10000 Zagreb, Croatia; martina.duras@vef.hr; 5 Laboratory for Mastitis and Raw Milk Quality, Department for Bacteriology and Parasitology, Croatian Veterinary Institute, Savska Cesta 143, 10000 Zagreb, Croatia; benic@veinst.hr

**Keywords:** essential trace elements, copper, zinc, tissues, stranded cetaceans, Adriatic Sea

## Abstract

Trace elements are widely distributed in the environment and are considered essential when their deficiency leads to impaired biological function. This study aimed to quantify concentrations of two essential trace elements—copper (Cu) and zinc (Zn)—in the tissues of three toothed whale (*Odontoceti*) species: bottlenose (*Tursiops truncatus*), striped (*Stenella coeruleoalba*), and Risso’s dolphins (*Grampus griseus*) found deceased along the Croatian coast of the Adriatic Sea between 1995 and 2013. A total of 190 individuals were analyzed, comprising 159 bottlenose, 25 striped, and 6 Risso’s dolphins. Concentrations of Cu and Zn were determined in liver, muscle, kidney, skin, lung, spleen, and fat tissues using inductively coupled plasma optical emission spectrometry (ICP-OES). The highest Cu concentrations were observed in the liver and kidneys of bottlenose dolphins, followed by striped and Risso’s dolphins. Zn concentrations were the highest in the skin of bottlenose and striped dolphins, whereas the liver exhibited the highest levels in Risso’s dolphins. In 14 bottlenose and 2 striped dolphins, Cu and Zn concentrations in liver tissue exceeded critical thresholds typically regulated by homeostatic mechanisms. Regression analysis indicated significant relationships between element concentrations, and both body length and body mass. In addition, trace element concentrations were positively correlated across individuals within the same tissue type, as well as among different tissues within the same individual. Overall, Cu and Zn concentrations exhibited a declining trend over the studied period across all tissue types. These findings provide important baseline data for future ecotoxicological investigations and contribute to conservation strategies for cetacean populations inhabiting the Adriatic Sea.

## Simple Summary

Trace elements are widespread in the environment and are considered essential when their absence leads to impaired biological function. This study investigated the concentrations of two essential trace elements—copper (Cu) and zinc (Zn)—in the tissues of three toothed whale (Odontoceti) species: bottlenose (*Tursiops truncatus*), striped (*Stenella coeruleoalba*), and Risso’s dolphins (*Grampus griseus*), found deceased in the Croatian part of the Adriatic Sea between 1995 and 2013. A total of 190 individuals were examined, comprising 159 bottlenose, 25 striped, and 6 Risso’s dolphins. Copper and zinc levels were quantified in liver, muscle, kidney, skin, lung, spleen, and fat tissues using inductively coupled plasma optical emission spectrometry (ICP-OES). Positive correlations in element concentrations were observed both across individuals within the same tissue type and between different tissues within the same individual. Overall, a decreasing trend in trace element concentrations was observed across all tissues over the study period. These results provide a valuable baseline for future toxicological and ecological studies, and contribute to the ongoing conservation efforts for dolphin populations in the Adriatic Sea.

## 1. Introduction

Following the Industrial Revolution, anthropogenic activities have significantly increased the release of various trace elements into the environment [1]. These elements are now widely distributed across ecosystems, and their presence in marine environments has become an area of growing scientific concern in recent decades [2].

Trace elements can be broadly classified into two categories: essential and non-essential. Essential trace elements are micronutrients required in small quantities for the optimal functioning of biological systems. Deficiency in these elements can impair physiological processes, as they are involved in a wide range of biological functions, including enzyme activity, protein transport, hormonal regulation, and receptor site functionality [3]. Notable essential trace elements include iron (Fe), zinc (Zn), manganese (Mn), copper (Cu), cobalt (Co), and selenium (Se), among others [4].

Conversely, non-essential trace elements are generally considered non-toxic when present below specific threshold levels. However, exceeding these thresholds can result in toxicological effects that disrupt critical physiological functions. Examples include chromium, silicon, and nickel. As noted by Roy [3], non-essential elements can be further categorized into non-toxic and toxic subgroups. Increasing attention is being directed toward the potential endocrine-disrupting effects of certain trace elements on marine mammals. Elements such as arsenic, cadmium, cobalt, chromium, copper, mercury, lead, selenium, and vanadium have been shown to interfere with estrogen, androgen, and glucocorticoid receptor-mediated processes both in vivo and in vitro, even at environmentally relevant concentrations. Therefore, it is imperative to assess trace element contamination in marine mammal tissues to evaluate their potential biological impacts [5].

Marine mammals occupy apex trophic levels within marine food webs and, due to their long lifespans and ecological roles, are considered valuable bioindicators for monitoring the spatial and temporal trends of environmental contaminants. Among them, the bottlenose dolphin (*Tursiops truncatus*) is particularly notable due to its widespread distribution, site fidelity to coastal habitats, well-documented life history, and demonstrated capacity for metal bioaccumulation, making it a suitable sentinel species for environmental monitoring [6]. In contrast, the striped dolphin (*Stenella coeruleoalba*) and Risso’s dolphin (*Grampus griseus*), also included in this study, are pelagic species that typically avoid shallow coastal waters. Given the biomagnification of metals through trophic transfer, top-level predators such as dolphins are likely to accumulate higher concentrations of trace elements. This trophic positioning, coupled with their ecological overlap with humans and shared dietary resources, underscores their relevance not only as indicators of marine ecosystem health, but also as proxies for human exposure risk [7].

Numerous studies have investigated trace element accumulation in dolphins across various geographic regions, including Japan [8,9], Australia [10,11,12,13], New Zealand [14], and the Atlantic coast [6,15,16]. In the Mediterranean Sea, research has predominantly focused on populations along the French [17,18], Italian [19,20], and Israeli coasts [21,22]. The bioaccumulation of trace elements in marine mammal tissues is influenced by multiple factors, including the geographic location, environmental contamination levels, age, and physiological condition of the individual [23].

The Adriatic Sea, a subregion of the Mediterranean, is home to a resident population of bottlenose dolphins (*Tursiops truncatus*), alongside occasional occurrences of other cetacean species [24]. Existing data on trace elements in dolphins from the Croatian part of the Adriatic are limited to few studies that focused primarily on non-essential trace elements in tissues of stranded bottlenose and striped dolphins as well as broader assessments of toxic element concentrations in these species and in Risso’s dolphins [25,26,27,28,29,30]. There are only two studies on the concentration of essential trace elements in the tissues of the mentioned dolphin species [31,32]. However, there remains a notable gap in comprehensive data encompassing a broader spectrum of trace elements. Establishing baseline concentrations of trace elements in cetacean tissues is critical for defining nutritional and toxicological reference values, enabling intra- and inter-regional comparisons, and facilitating the linkage of contaminant burdens to observed mortality events.

The present study aimed to quantify concentrations of two essential trace elements—copper (Cu) and zinc (Zn)—in various tissues of dolphins stranded along the Croatian Adriatic coastline between 1995 and 2013. These elements were selected due to their essential biological roles and their potential to exert toxic effects at elevated concentrations [33]. The study also aimed to compare the obtained values with data from global cetacean populations.

This research contributes to the understanding of trace element dynamics in Adriatic dolphin populations, which have been under systematic observation for over three decades. Given that tissue samples are obtained post-mortem, often from stranded individuals, the consistent monitoring of deceased specimens can serve as an important tool for tracking contaminant trends, evaluating animal health, and identifying the causes of unusual mortality events.

## 2. Materials and Methods

### 2.1. The Study Area and Sample Collection

**Figure 1:**
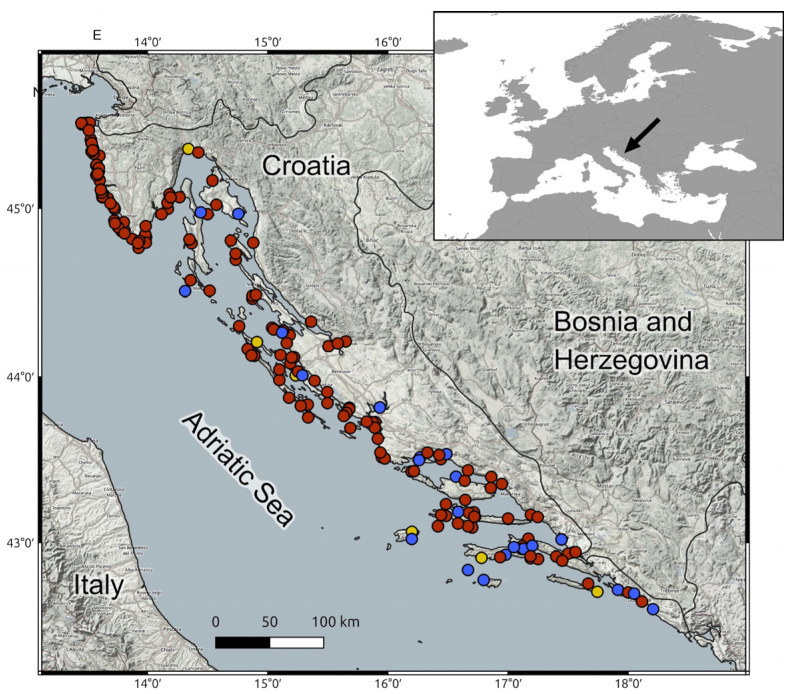
Geographical distribution of stranded cetaceans analyzed in this study, recorded along the Croatian Adriatic coastline between 1995 and 2013. RED—bottlenose dolphins (*Tursiops truncatus*), BLUE—striped dolphin (*Stenella coeruleoalba*), YELLOW—Risso dolphin (*Grampus griseus*).

Tissue samples were collected from a total of 190 dolphins found stranded and deceased along the Croatian coastline of the Adriatic Sea between 1995 and 2013. The specimens comprised 159 bottlenose dolphins (*Tursiops truncatus*), 25 striped dolphins (*Stenella coeruleoalba*), and 6 Risso’s dolphins (*Grampus griseus*) (Figure 1).

As part of the post-mortem examination, a range of biological and morphometric data were recorded, including species, sex, body mass, total body length, and other standard external measurements. Age determination was conducted by preparing tooth samples according to the methodology outlined by Slooten, with age estimated through the enumeration of growth layer groups (GLGs) as described by Hohn et al. Individuals older than 7 years were classified as adults, based on criteria established by André et al. [34,35,36].

As summarized in Table 1, bottlenose dolphin samples were classified by sex (77 males, 81 females) and age (77 adults, 52 juveniles). Age and sex could not be determined for 30 and one bottlenose dolphins, respectively. Striped dolphins were categorized solely by sex due to missing age data, comprising 15 males and 10 females. The six Risso’s dolphin samples were all adults; sex was not specified owing to the limited sample size.

Tissue samples from these dolphins had previously been partly analyzed for copper concentrations using graphite furnace atomic absorption spectroscopy (GFAAS), as reported by Bilandžić et al. [31]. In the present study, those same specimens were reanalyzed to quantify copper levels using an advanced analytical method described later in the text.

For spatial analysis, the Croatian Adriatic was divided into two geographical regions—northern and southern—with a higher proportion of samples originating from the southern region. The mean (±standard deviation) body lengths and masses were as follows: bottlenose dolphins, 239.2 ± 53.9 cm and 150.5 ± 79.8 kg; striped dolphins, 191.5 ± 40.3 cm and 81.1 ± 15.4 kg; and Risso’s dolphins, 300.1 ± 10.6 cm and 258.8 ± 46.4 kg.

During necropsy, tissue samples were collected from various organs and tissues including muscle, liver, kidneys, spleen, lungs, blubber, and skin. Samples were stored in polyethylene bags and preserved at −20 °C until further analysis.

### 2.2. Sample Preparation and Trace Element Determination

Tissue samples (0.5 g) were digested using microwave-assisted digestion in Teflon vessels. The digestion mixture comprised 2.5 mL of nitric acid (65%), 1.0 mL of hydrogen peroxide (30%) provided from Merck Suprapur^®^ (Merck KGaA, Darmstadt, Hesse, Germany), and 3.0 mL of ultrapure water. Following digestion and cooling, each sample was diluted to a final volume of 50 mL with ultrapure water in a volumetric flask. Element concentrations were expressed in milligrams per kilogram (mg/kg) on a wet weight basis. For comparative purposes with data from other studies reported on a dry weight basis, values were converted to wet weight using a standard conversion factor of 0.25 [37].

Copper (Cu) and zinc (Zn) concentrations were quantified using an inductively coupled plasma optical emission spectrometer (ICP-OES), Model Optima 8000, equipped with an S10 autosampler (PerkinElmer, Waltham, MA, USA). Quality assurance and quality control (QA/QC) procedures included the use of analytical blanks, replicate measurements, and certified reference materials (CRMs), specifically DORM-4 and DOLT-5, obtained from the National Research Council (NRC), Institute for National Measurement Standards, Ottawa, ON, Canada. The recovery rates for the CRMs, as well as the instrumental quantification limits for each element, are presented in Table 2.

### 2.3. Statistical Analysis

All statistical analyses were performed using Stata version 13.1 (StataCorp, College Station, TX, USA). For each trace element, descriptive statistics—including the mean, standard deviation, median, minimum (m), and maximum (M) values—were calculated. The Shapiro–Wilk test was used to assess the normality of the data distribution for element concentrations across tissue types. Comparisons of element concentrations among the three dolphin species were conducted using the non-parametric Kruskal–Wallis test, and statistical significance was reported using *p*-values.

To investigate the linear relationships between the logarithm of element concentrations and independent variables such as body mass, body length, age, sex, sampling location, and year of discovery, linear regression analysis was employed. The regression model followed the equation log*c* = log*b* × *x* + *a*, where *c* denotes the mass concentration of the element in the tissue [mg/kg], *a* is the constant intercept, log *b* is the slope of the line (regression coefficient), and *x* is the independent variable.

The tables presented in this study display the antilogarithmic values of the regression coefficients, denoted as b. These coefficients represent the extent of change in the mass concentration when the independent variable, *x*, undergoes a unit change. A value of *b* greater than 1 indicates an increase in concentration with the variable, while a value less than 1 suggests a decrease. To assess the relationships between element concentrations within the same tissue type across individuals, and between different tissues within the same individual, Spearman’s rank correlation coefficient was used. The strength of correlations was interpreted using a commonly accepted gradation scale (0.00 to 0.19—very weak, 0.20 to 0.39—weak, 0.40 to 0.59—moderate, 0.60 to 0.79—strong, and 0.80 to 1.0—very strong).

## 3. Results

Concentrations of essential trace elements in the tissues of three dolphin species stranded along the Croatian coast of the Adriatic Sea between 1995 and 2013 are presented in Table 3. Values are expressed as the mean ± standard deviation (SD) on a wet weight basis. For comparative purposes, manganese concentrations previously determined in the same individuals and published by Đokić et al. [32] are included in the final column, allowing for comparison with copper and zinc concentrations.

### 3.1. Copper Levels in the Tissues of Three Dolphin Species

In bottlenose dolphins, the highest copper (Cu) concentrations were observed in the following order: liver > kidney > muscle > lung > spleen. For striped dolphins, the order was liver > kidney > muscle > spleen > lung, while in Risso’s dolphins, the sequence was liver > kidney > spleen > lung > muscle. Statistically significant interspecific differences in Cu concentrations were detected in the liver (*p* = 0.0030), kidney (*p* = 0.0077), and lung (*p* = 0.0010), with bottlenose dolphins exhibiting the highest concentrations in all three tissues.

**Figure 2:**
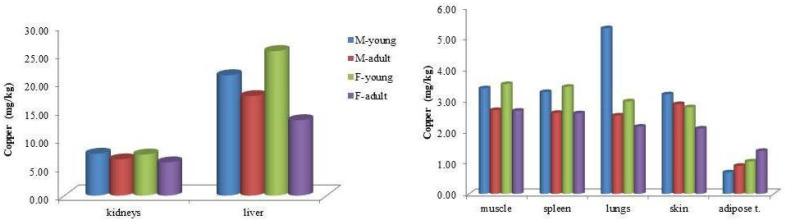
Mean copper (Cu) concentrations (mg kg^−1^ wet weight) in the tissues of bottlenose dolphins (*Tursiops truncatus*), categorized by sex and age class.

Analysis of Cu concentrations by age and sex within bottlenose dolphins revealed generally similar values across categories, with exceptions in liver and lung tissues. In the liver, adult males exhibited higher Cu concentrations than adult females (17.6 vs. 13.3 mg/kg, respectively). Conversely, among juveniles, females showed higher hepatic Cu levels than males (24.9 vs. 21.3 mg/kg). In lung tissue, juvenile males had elevated Cu concentrations compared to juvenile females (5.32 vs. 2.97 mg/kg, respectively; Figure 2).

**Figure 3:**
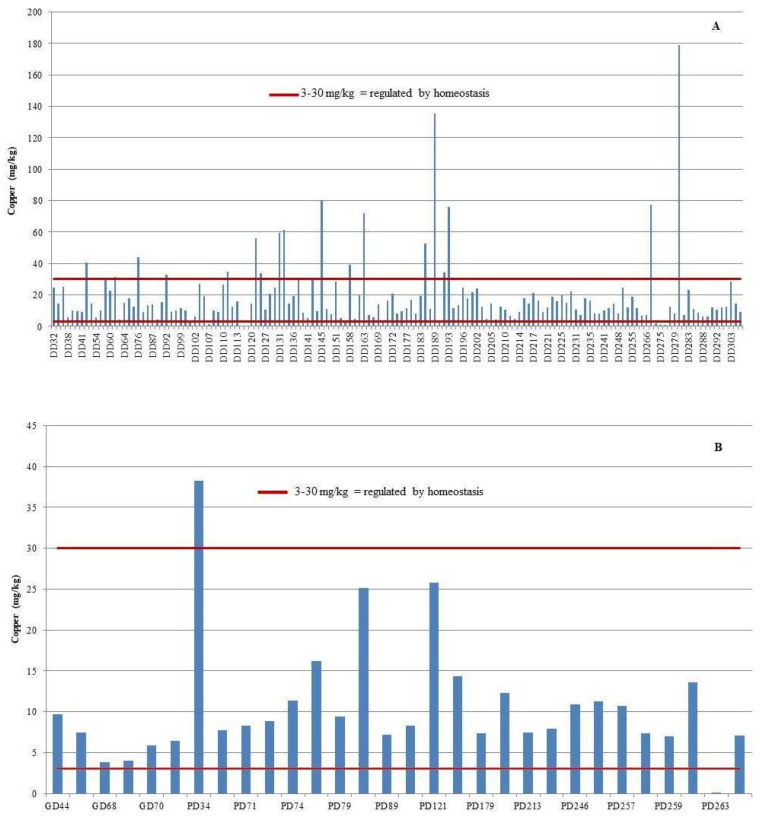
Individual copper (Cu) concentrations in the liver of bottlenose dolphins (*Tursiops truncatus*, DD) (**A**), striped dolphins (*Stenella coeruleoalba*, PD), and Risso’s dolphins (*Grampus griseus*, GD) (**B**), shown in comparison to established critical concentration thresholds.

The liver exhibited the highest copper (Cu) concentrations across all examined tissues, with bottlenose dolphins showing the greatest mean hepatic Cu level at 18.9 mg/kg wet weight (range: 0.001–178.9 mg/kg). Striped dolphins followed with a mean of 11.8 mg/kg (range: 0.001–38.2 mg/kg), while Risso’s dolphins exhibited the lowest hepatic Cu concentrations, averaging 6.20 mg/kg (range: 3.81–9.69 mg/kg). The highest individual Cu concentration was detected in the liver of a female bottlenose dolphin stranded near the island of Ist, reaching 178.86 mg/kg wet weight (Figure 3).

### 3.2. Zinc Levels in the Tissues of Three Dolphin Species

**Figure 4:**
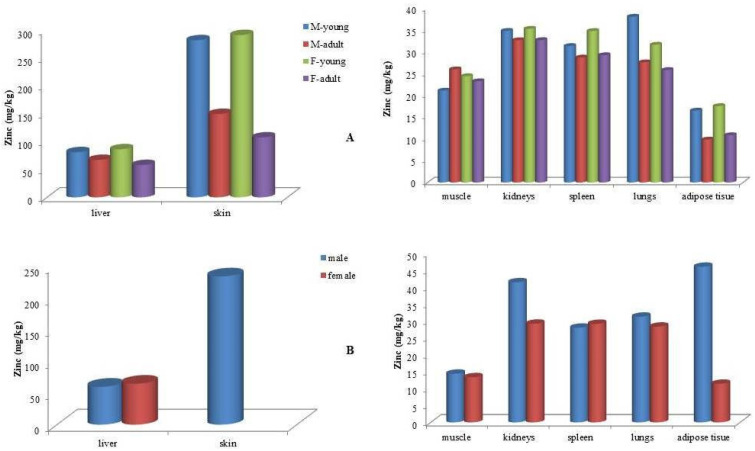
Mean zinc (Zn) concentrations (mg kg^−1^ wet weight) in tissues of (**A**) bottlenose dolphins (*Tursiops truncatus*) and (**B**) striped dolphins (*Stenella coeruleoalba*) categorized by sex and age class.

Zinc (Zn) concentrations in all examined tissues across the three dolphin species followed a consistent pattern: skin > liver > kidney > other organs (Table 2). The highest Zn concentrations in skin were observed in juvenile bottlenose dolphins, with mean values of 291.1 mg/kg in females and 281.7 mg/kg in males. In comparison, male striped dolphins exhibited slightly lower mean skin Zn concentrations (233.5 mg/kg). The lowest skin Zn levels were recorded in adult bottlenose dolphins, with 149.4 mg/kg in males and 107.3 mg/kg in females (Figure 4).

The maximum Zn concentration (462.9 mg/kg) was detected in the skin of a female bottlenose dolphin of unknown age stranded near the island of Brač. Similarly high levels were measured in a 6-year-old female from open waters in the southern Adriatic (446.8 mg/kg).

Statistically significant interspecific differences in Zn concentrations were observed in muscle (*p* = 0.0001) and lung tissues (*p* = 0.0024). Risso’s dolphins exhibited the highest muscle Zn concentrations, while bottlenose dolphins had the highest levels in the lungs.

Regarding liver tissue, the highest mean Zn concentrations were found in juvenile bottlenose dolphins, with 86.1 mg/kg in females and 80.9 mg/kg in males. These values were followed by adult males (67.0 mg/kg) and adult females (57.9 mg/kg) (Figure 4). In striped dolphins, liver Zn levels averaged 59.7 mg/kg in males and 64.5 mg/kg in females. The lowest mean liver Zn concentration (46.2 mg/kg) was observed in Risso’s dolphins.

**Figure 5:**
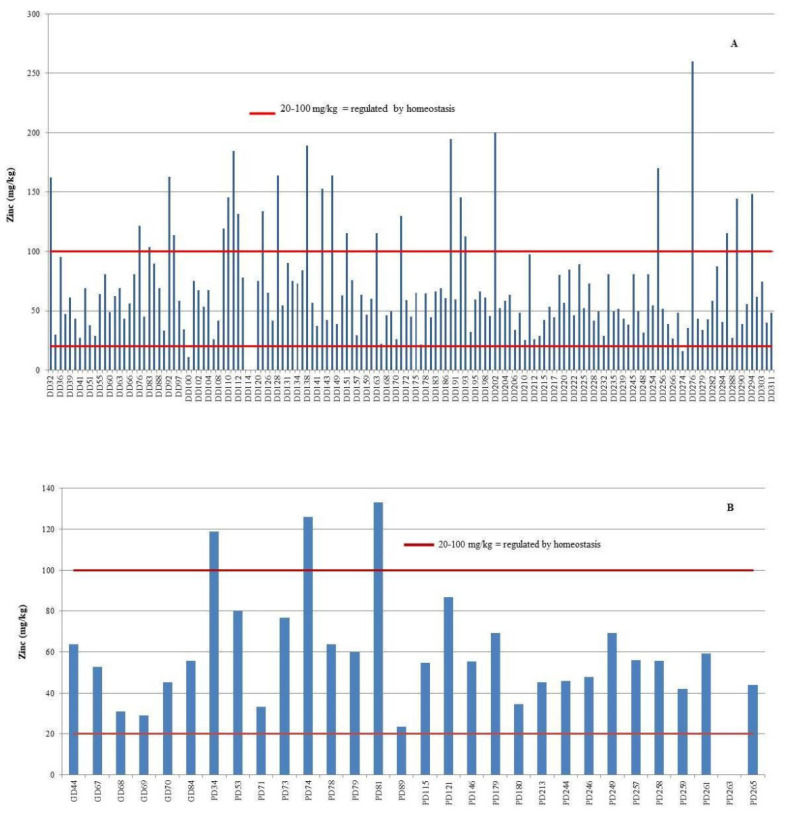
Individual zinc (Zn) concentrations in the liver of bottlenose dolphins (*Tursiops truncatus*, DD) (**A**), striped dolphins (*Stenella coeruleoalba*, PD), and Risso’s dolphins (*Grampus griseus*, GD) (**B**), shown in comparison to established critical concentration thresholds.

Zinc (Zn) concentrations in the liver exceeded the established homeostatic range in 33 individuals, comprising 29 bottlenose dolphins (18.4%) and 4 striped dolphins (16%) (Figure 5). Among these, hepatic Zn concentrations surpassed the upper critical threshold of 100 mg/kg in 26 bottlenose dolphins (16.5%) and 3 striped dolphins (12%). Conversely, concentrations fell below the lower critical limit of 20 mg/kg in three bottlenose dolphins (1.9%) and one striped dolphin (4%).

Of the 12 dolphins diagnosed with congenital umbilical hernia as the cause of death, 4 individuals (33.3%) exhibited elevated hepatic Zn concentrations. In contrast, none of the individuals that died as a result of severe parasitic infestation presented with reduced Zn levels in the liver.

### 3.3. Influence of Biological and Environmental Variables on Trace Element Concentrations (Regression Analysis)

Regression analysis revealed that the year of stranding significantly influenced the concentrations of copper (Cu) and zinc (Zn) in dolphin tissues. A statistically significant inverse relationship was found between the year of stranding and Cu levels in the skin (*p* = 0.030, *b* = 0.604), as well as Zn concentrations in the kidney (*p* = 0.018, *b* = 0.977), liver (*p* = 0.024, *b* = 0.943), and spleen (*p* = 0.018, *b* = 0.945). These findings indicate a general temporal decline in trace element concentrations across the study period.

Geographic location also influenced trace element levels. Specifically, Cu concentrations in muscle tissue were significantly lower in dolphins stranded in southern regions compared to those from northern areas (*p* = 0.049, *b* = 0.803).

Age was found to be significantly associated with Cu levels in the lungs (*p* = 0.034, *b* = 0.968), with concentrations decreasing as age increased.

Body mass was inversely correlated with Cu concentrations in the kidney (*p* = 0.009, *b* = 0.997) and adipose tissue (*p* = 0.018, *b* = 0.986), as well as Zn levels in the kidney (*p* < 0.001, *b* = 0.996). In contrast, an increase in body length was associated with elevated Cu concentrations in the kidney (*p* = 0.033, *b* = 1.003) and adipose tissue (*p* = 0.046, *b* = 1.018).

### 3.4. Spearman’s Correlation

Spearman’s rank correlation coefficient was employed to assess the relationships between trace element concentrations across different tissues, as well as within the same tissue in relation to species, sex, and age.

Strong positive correlations for copper (Cu) were observed between the kidney and spleen (*r^s^* = 0.6571; *p* < 0.05), spleen and adipose tissue (*r^s^* = 0.6595; *p* < 0.05), and lung and adipose tissue (*r^s^* = 0.6036; *p* < 0.05) across all three dolphin species. Similarly, zinc (Zn) showed strong positive correlations between the kidney and spleen (*r^s^* = 0.6370; *p* < 0.05), and between the kidney and lung (*r^s^* = 0.6134; *p* < 0.05). The strongest correlation in the dataset was observed between Cu concentrations in the spleen and lungs (*r^s^* = 0.8038; *p* < 0.05).

Moderate positive correlations were noted between Cu and Zn concentrations in the liver (*r^s^* = 0.5050; *p* < 0.05) and kidney (*r^s^* = 0.5639; *p* < 0.05), indicating a degree of co-accumulation or similar regulatory patterns for these elements in these tissues.

Sex-related differences in element distribution were also evident. In female dolphins, a strong correlation was found between Cu levels in the kidney and liver (*r^s^* = 0.6713; *p* < 0.05). Furthermore, a significant influence of sex was detected on the correlation between Cu and Zn concentrations in the kidneys of male bottlenose and striped dolphins (*r^s^* = 0.6179; *p* < 0.05).

## 4. Discussion

### 4.1. Copper

This study supports the well-established role of the liver as the principal organ for copper (Cu) accumulation in marine mammals [38]. Among the three species examined, bottlenose dolphins exhibited the highest hepatic Cu concentrations (mean: 18.9 mg/kg; range: 0.001–178.9 mg/kg), followed by striped dolphins (11.8 mg/kg) and Risso’s dolphins (6.20 mg/kg). These findings are consistent with previously observed patterns of trace element storage in cetaceans, where the liver serves as a key site for metal detoxification and regulation [39,40].

Table 4 presents the concentrations of copper (Cu) for the studied species analysed in the Mediterranean and other geographic regions worldwide. Values are presented as mean concentrations on a wet weight basis.

#### 4.1.1. Copper Concentration Across Tissues

Liver: Hepatic Cu concentrations in bottlenose dolphins were notably higher than previously reported in the Mediterranean. For example, dolphins from the Ligurian Sea had values of 14.61 mg/kg (range: 5.45–23.78 mg/kg) [20], and those from Israel ranged from 1 to 30 mg/kg with a mean around 11.4–11.8 mg/kg [44,51]. The concentration observed in striped dolphins (11.8 mg/kg) also ranks among the highest in the Mediterranean, aligning with values off the Israeli coast [51], and exceeding typical levels (<10 mg/kg) found elsewhere. In Risso’s dolphins, hepatic Cu concentrations (6.20 mg/kg) were consistent with those from Israel (6.11 and 6.49 mg/kg) [21,52], but higher than in individuals from the Ligurian Sea (2.58 mg/kg) [20], Corsican coast (2.5 mg/kg) [42], and Italian coast (2.44 mg/kg) [41]. These interspecific differences may reflect dietary, physiological, and ecological variability among species [2,41].

Globally, hepatic Cu concentrations in bottlenose dolphins have shown considerable variability. Reported mean values include 10.78 mg/kg in South Carolina, USA (range: 1.17–78.98 mg/kg) [33], 21.18 mg/kg in southern Australia (range: 26.16–68 mg/kg) [12], and 17.75 mg/kg in Texas (range: 1.98–54.25 mg/kg) [57]. Exceptionally high concentrations have been reported in the Atlantic off the Portuguese coast, reaching up to 11,982 mg/kg (range: 4756–30,636 mg/kg) [61].

Kidney: Significant interspecies differences in renal Cu levels were identified (*p* < 0.05). Bottlenose dolphins exhibited the highest kidney concentrations (6.26 mg/kg), followed by striped (5.43 mg/kg) and Risso’s dolphins (3.52 mg/kg). This pattern parallels hepatic Cu distribution. No significant differences were found across age or sex in bottlenose dolphins, though adult females showed the broadest range (0.001–31.3 mg/kg). In striped dolphins, males had slightly higher levels (6.05 mg/kg) than females (4.56 mg/kg). Compared with external populations, renal Cu levels in bottlenose dolphins matched those in the Ligurian Sea [20] but were lower than those from Texas (12 mg/kg) and southern Australia (7.35 mg/kg) [12,57]. Interestingly, kidney Cu in striped dolphins was consistently higher than in previous Mediterranean reports, suggesting possible regional differences in exposure or retention.

Lung: The highest pulmonary Cu level (61.9 mg/kg) was found in a young male bottlenose dolphin (mean: 2.88 mg/kg). Adult males, juveniles, and adult females showed similar values (~2–3 mg/kg). In contrast, striped and Risso’s dolphins had markedly lower lung Cu levels (mean: ~1.4–1.5 mg/kg). Bottlenose dolphin lung values were comparable to those from Corsica (2.25 mg/kg) [42] but exceeded most other Mediterranean values, generally <1 mg/kg, except in the Ligurian Sea (1.41 mg/kg) [20], southern Italy (1.20 mg/kg) [47], and central Italy (1.09 mg/kg) [41].

Spleen, skin and adipose tissue: Few data exist for these tissues in the Mediterranean. In this study, spleen Cu concentrations across all species exceeded previous records. In skin, bottlenose dolphins averaged 2.54 mg/kg (range: 0.17–15.4 mg/kg), exceeding values from Corsica (0.85 mg/kg) [42], Israel (1.1 mg/kg) [22], and comparable to Portugal (2.13 mg/kg) [55], but lower than the peak average reported in Israel (4.73 mg/kg) [21]. Striped dolphin skin Cu levels (1.99 mg/kg) also aligned with Atlantic data from Portugal (1.95 mg/kg) [55] and exceeded most Mediterranean values, aside from Israel (2.1 mg/kg) [22]. In adipose tissue, bottlenose dolphins (1.47 mg/kg) showed higher Cu concentrations than previously reported in Israel (0.36 mg/kg) [22]. Striped dolphins, however, exhibited lower fat Cu levels (0.49 mg/kg) than those reported in most other Mediterranean studies [19,22,47,51].

#### 4.1.2. Age-Related Patterns

A pronounced age-related trend was observed. A newborn bottlenose dolphin from Apulia, Italy, showed an exceptionally high hepatic Cu concentration (80.65 mg/kg), in contrast to a juvenile (19.34 mg/kg) and an adult female (8.29 mg/kg) from the same region [63]. This decline with age mirrors patterns reported in striped dolphins from the French coast [48], suggesting a consistent trend across species and regions. Elevated hepatic Cu in neonates is well-documented and attributed to transplacental transfer, coupled with limited excretory ability during early development [8,64,65,66,67,68]. This accumulation may be facilitated by high fetal metallothionein expression, which aids Cu storage during gestation [69]. Since mammalian milk contains minimal Cu [70], prenatal accumulation is essential to support postnatal growth. Importantly, copper regulation in cetaceans is considered tightly maintained, with variation occurring primarily during early developmental stages [71,72,73].

#### 4.1.3. Dietary Influence and Bioaccumulation

Diet plays a central role in Cu accumulation [19,21,41,57]. Risso’s dolphins off Israel showed liver Cu levels reflecting high Cu in their preferred prey, cuttlefish (*Sepia officinalis*)—up to 1450 mg/kg [21]. Similarly, cephalopods from the stomachs of Corsican striped and Risso’s dolphins were Cu-rich [42]. In the Adriatic, bottlenose dolphins primarily consumed fish (e.g., *Conger conger*, *Merluccius merluccius*, *Pagellus erythrinus*) and squid (*Loligo vulgaris*), recognized Cu sources in the region [74].

#### 4.1.4. Toxicological Implications

Establishing reference values is essential to assess potential Cu toxicity. Law et al. [53] proposed a hepatic Cu range of 3–30 μg/g wet weight as physiologically regulated. Concentrations exceeding this range suggest impaired homeostatic control. In this study, several individuals—particularly young dolphins—exceeded the upper threshold, as illustrated in Figure 3, underscoring the need for further monitoring of vulnerable age groups and polluted habitats.

### 4.2. Zinc

#### 4.2.1. Zinc Concentration Across Tissues

Table 5 presents the concentrations of zinc (Zn) for the studied species analysed in the Mediterranean and other geographic regions worldwide. Values are presented as mean concentrations on a wet weight basis.

Liver: Hepatic Zn concentrations in bottlenose dolphins aligned with those reported in individuals from the Ligurian Sea [20] and the Italian coast [41]. Striped dolphins showed hepatic Zn levels comparable to those reported from the Italian [41] and Israeli coasts [51], with mean concentrations up to twice as high as in dolphins from southern Italy [19], the French coast [43], and the Corsican coast [42]. The concentrations in all three species examined in this study are consistent with values reported for various global locations, including the Atlantic coast of Portugal, Florida, Hawaii, South Carolina, Texas, Japan, and England.

Kidney: In kidney tissue, bottlenose and striped dolphins exhibited broader Zn concentration ranges than those documented in the Mediterranean. In bottlenose dolphins, renal Zn concentrations ranged from 0.03 to 92 mg/kg, while in striped dolphins, concentrations ranged from 23.1 to 109.8 mg/kg. In the Mediterranean region, the widest concentration range was observed in dolphins from the Israeli coast, where bottlenose dolphins had renal Zn values ranging from 13 to 30 mg/kg and striped dolphins from 17 to 51 mg/kg [22]. In the French coast, striped dolphins had a mean kidney Zn concentration of 34.88 mg/kg [43], aligning with this study’s mean of 36.3 mg/kg. Zn concentrations in Risso’s dolphins were consistent with findings from Israel [21,52], Italy [41], and Corsica [42].

Muscle and spleen: Muscle tissue of bottlenose dolphins showed a mean Zn concentration of 23.7 mg/kg, similar to individuals from the Ligurian Sea [20] and the Israeli coast [22,44], but approximately twice the levels found in individuals from the Italian [41] and Corsican coasts [42]. In striped dolphins, Zn concentrations in muscle ranged from 6.49 to 38.1 mg/kg, values that match those observed in the Ligurian Sea [45], Israeli coast [51], and French coast [48]. The highest range in the Mediterranean was recorded in striped dolphins from the Israeli coast (8.5–60.1 mg/kg, mean 23.2 mg/kg) [51]. Lower values were found in individuals from the Corsican coast (6 mg/kg) [42]. In Risso’s dolphins, muscle Zn levels reached 25.0 mg/kg in this study, double the values found in Italian specimens (10.25 mg/kg) [41], but lower than those reported in Israeli dolphins (68.8 mg/kg) [21].

In the spleen, Zn concentrations in striped dolphins were consistent with those reported for the Ligurian Sea [20,45] and Japan [62].

#### 4.2.2. Factors Affecting Zinc Concentrations

Zn predominantly accumulates in the skin of marine mammals, a pattern consistent with its physiological roles in wound healing and photoprotection from ultraviolet radiation [76]. Aubail et al. [55] demonstrated that Zn concentrations are significantly higher in the upper adipose layer compared to the middle and deepest layers, further supporting its role in surface-level protection.

When comparing Zn concentrations in the skin of all three dolphin species, only individuals from the Israeli coast exhibited significantly higher values compared to other Mediterranean locations. Zn concentrations in skin tissues from dolphins in this study were similar to those found in dolphins elsewhere (Table 5), with particularly high mean concentrations recorded in bottlenose dolphins (553.3 mg/kg; range: 336.3–1202 mg/kg) and striped dolphins (526 mg/kg; range: 252.3–1095 mg/kg) from the Portuguese Atlantic coast [55]. However, skin Zn levels in bottlenose dolphins from the same location but during a different period were much lower (mean: 45 mg/kg; range: 20–55.5 mg/kg) [54]. Similar temporal variations were observed in adipose tissue, where bottlenose dolphins had mean Zn values of 5.8 mg/kg between 1998 and 2002, and 52.5 mg/kg during 2001–2008 [54,55].

In the adipose tissue of bottlenose and striped dolphins from this study, Zn concentrations exceeded those recorded in other parts of the Mediterranean. Additionally, Zn levels varied based on age and sex. Juvenile bottlenose dolphins exhibited higher concentrations than adults, with females showing mean levels of 17.5 mg/kg and males 16.5 mg/kg, compared to 10.8 mg/kg in adult females and 9.83 mg/kg in adult males. Comparable values were found in dolphins from West Wales and the Portuguese Atlantic coast [55,56].

#### 4.2.3. Zinc Concentrations and Health Status

Elevated hepatic Zn concentrations have been linked to disease states in cetaceans. For instance, in coastal harbor porpoises (*Phocoena phocoena*), individuals that died from infectious diseases had significantly higher Zn levels in liver tissue than those that succumbed to physical trauma [77]. This suggests a possible redistribution or retention of Zn in response to infection, potentially due to its role in immune function.

### 4.3. Limit Concentrations of Copper and Zinc

Analysis of hepatic copper (Cu) and zinc (Zn) concentrations in 16 dolphin samples revealed values outside the physiologically regulated homeostatic ranges (Cu: 3–30 mg/kg; Zn: 20–100 mg/kg) [53]. Specifically, elevated or reduced concentrations were identified in 14 bottlenose dolphins (*Tursiops truncatus*)—representing 8.9% of the total bottlenose dolphin samples—and in 2 striped dolphins (*Stenella coeruleoalba*), comprising 8% of that species group.

Among the 14 bottlenose dolphins with values outside the normal range, 6 were males (42.9%) and 8 were females (57.1%). Both striped dolphins exhibiting abnormal values were males. In 12 of the 16 individuals—11 bottlenose dolphins and 1 striped dolphin—concentrations of both Cu and Zn exceeded the upper critical threshold. These 12 individuals were evenly distributed between sexes: 6 males and 6 females. All female dolphins in this subset were bottlenose dolphins; among the males, 5 were bottlenose and 1 was a striped dolphin. Elevated Cu and Zn concentrations in some individuals occasionally exceeded critical thresholds for physiological homeostasis, raising concerns about health risks such as impaired metabolic function and increased disease susceptibility. These findings highlight the need for further research into the links between trace element accumulation, immune function, and disease in dolphin populations. Notably, all 12 individuals exhibiting supra-physiological concentrations of both metals were found in the southern Adriatic Sea, suggesting potential spatial correlation with localized anthropogenic pollution sources, as previously reported [53].

Of the remaining four individuals, three dolphins (two bottlenose and one striped) exhibited hepatic Cu and Zn concentrations below the critical threshold values (Cu < 3 mg/kg; Zn < 20 mg/kg). One bottlenose dolphin displayed the lowest Cu concentration measured in this study (1.014 mg/kg), while simultaneously exhibiting the highest hepatic Zn concentration recorded (259.81 mg/kg), greatly exceeding the upper homeostatic range.

A positive correlation was observed between Cu and Zn concentrations in the liver, likely attributable to their co-binding affinity for metallothionein proteins [46,78,79]. Similar patterns were reported in a study on mass mortality events in Caspian seals (*Phoca caspica*), where certain individuals demonstrated elevated hepatic Zn levels concurrent with abnormally low Cu concentrations [80]. One potential mechanism involves dietary interference, whereby excessive Zn or iron intake disrupts intestinal Cu absorption through competitive inhibition at the mucosal level, resulting in hepatic Cu depletion [81].

### 4.4. Influence of Spatio-Temporal Factors, Sex, Age, and Morphometric Parameters on Trace Element Concentrations

Regression analysis revealed a statistically significant temporal trend in the concentrations of copper (Cu), manganese (Mn), and zinc (Zn) in dolphin tissues over the study period, indicating a general decrease in all three elements across years. These findings are consistent with the report by Zhou et al. [82], who observed temporal variations in trace element accumulation, including an increase in Cu concentrations in the muscle of common dolphins (*Delphinus delphis*) from the Atlantic coast of Portugal between 1995 and 1997, followed by a decrease in 1998. During that period, decreases in hepatic Zn and Cu, as well as renal Cu concentrations, were also documented. Mn levels in the liver fluctuated, while Zn concentrations in the kidney and muscle remained relatively stable [82]. Conversely, studies on the same species from the French Atlantic coast reported no significant differences in Cr, Cu, or Zn concentrations between 1977–1980 and 1984–1990 [43].

Data from the French Mediterranean coast indicated stable Cu and Zn concentrations in the tissues of striped dolphins between 2002 and 2009 [48]. Similarly, in the Eastern Mediterranean (Levantine Basin), no significant temporal variations in Cu, Mn, or Zn concentrations were detected in bottlenose dolphins between 2004 and 2006 [44] or 1994 and 2001 [22], nor in striped dolphins from Israel between 2001 and 2011 [51]. The exception was hepatic Mn, which was significantly higher in 2006–2011 compared to 2001. These findings support the notion that trace element pollution in the Mediterranean has either stabilized or declined over the past decade. However, as Wafo et al. [48] emphasized, a reduction in environmental inputs does not immediately translate into decreased concentrations within marine organisms due to the persistence and bioaccumulation properties of trace elements. A longer temporal frame of approximately two decades, as considered in this study, reveals a clearer trend of declining concentrations.

In contrast, a comparative study of hepatic Cu concentrations in striped dolphins from the western Mediterranean and the Atlantic Ocean reported no significant differences [50]. Regional variations in cobalt concentrations in the skin of bottlenose dolphins from Florida and South Carolina were documented by Stavros et al. [58], highlighting spatial variability. Although anthropogenic emissions of trace elements are lower in the Southern Hemisphere [83], higher concentrations have been observed in marine organisms from this region, including areas like the Southern Ocean and the South Pole, which are generally regarded as less contaminated [84]. These discrepancies suggest that tissue concentrations of trace elements do not necessarily mirror environmental contamination levels. Instead, the trace element content in marine mammal tissues is more strongly influenced by factors such as bioavailability and dietary intake [85,86,87].

Sex-related differences in trace element concentrations were not detected in this study for Cu or Zn in any tissue. However, consistent with earlier research, Mn concentrations in the kidney were significantly lower in females compared to males (*p* = 0.008, *b* = 0.850), a trend also noted by Monteiro et al. [61]. Other authors reported no influence of sex on trace element levels [49,50,60,82].

A significant correlation between age and trace element concentration was observed for Cu in the lung (*p* = 0.034, *b* = 0.968), with levels declining as individuals aged. Higher concentrations of Cu and Zn were found in juveniles, including foetuses and neonates, compared to adults. These findings align with previously published studies [5,6,48,58,60,62] and support the understanding that essential trace elements accumulate during periods of rapid tissue development and cellular differentiation. Elevated Cu and Zn levels in young individuals may reflect both increased physiological demand and limited excretion, particularly in fetal tissues [2].

Body length and mass significantly influenced the concentration of trace elements in various tissues, although no consistent pattern was evident across all elements. Specifically, Cu concentrations in the kidney and adipose tissue, and Mn in the kidney (*p* = 0.013, *b* = 0.998), liver, and adipose tissue, as well as Zn in the kidney, decreased with increasing body weight. Conversely, Cu concentrations in the kidney and adipose tissue increased with greater body length (*p* = 0.046, *b* = 1.018). These relationships suggest that metabolic regulation and transfer of essential elements have a more pronounced impact on trace element content in tissues than chronological age or cumulative environmental exposure [75].

### 4.5. Relationships Between Trace Element Concentrations

Numerous studies have reported positive correlations in the concentrations of identical trace elements across different tissues of marine mammals. Consistent with these findings, the present study demonstrated uniformly positive correlations between tissues for all examined elements, corroborating results from previous investigations [22,55,88].

Copper (Cu) and zinc (Zn) are essential trace elements involved in the structure and catalytic activity of numerous metalloenzymes. Their homeostasis is regulated by metallothioneins, a class of low-molecular-weight, cysteine-rich proteins with high binding affinity for divalent metal ions [89]. Given this shared regulatory mechanism, an antagonistic relationship between Cu and Zn might be expected, as suggested by earlier literature [90]. However, in this study, as in that of Lemos et al. [59], a statistically significant positive correlation was observed between Cu and Zn concentrations.

This finding suggests the presence of additional, yet unidentified, mechanisms that modulate the relationship between Cu and Zn in cetacean tissues. The observed correlations are likely mediated by complex interactions involving both biotic factors (e.g., diet, age, sex, physiological condition) and abiotic variables (e.g., geographic location, environmental contamination) rather than a direct biochemical antagonism [91,92,93]. Consequently, further targeted studies are necessary to elucidate the nature of these interactions and their potential physiological or ecological implications

## 5. Conclusions

This study assessed copper (Cu) and zinc (Zn) concentrations in 190 samples from stranded dolphins collected along the Croatian Adriatic coast between 1995 and 2013. When compared with data from other regions of the Mediterranean Sea, the concentrations of Cu and Zn in these specimens were generally elevated. Notably, Zn levels in the skin were consistent with existing findings that highlight the skin as a preferential site of Zn accumulation in marine mammals.

In 16 individuals, hepatic concentrations of Cu and Zn were found to be outside the homeostatically regulated range. Among these, 12 individuals—located exclusively in the southern Adriatic—exhibited Cu and Zn concentrations above the upper critical thresholds, suggesting localized environmental contamination as a likely source of exposure. These findings reinforce the utility of liver tissue as a biomarker of chronic trace metal exposure in marine mammals.

Temporal analysis revealed a statistically significant decline in trace element concentrations over the nearly two-decade study period. This trend may result from changes in environmental conditions, improved pollution control or shifts in the dolphins’ feeding habits. The causes and ecological impacts of this trend need further investigation to assess its relevance to dolphin health and habitat quality. Among the various tissue types, liver and kidney exhibited the strongest positive correlations between Cu and Zn concentrations. This observation challenges the widely held notion of a strictly antagonistic relationship between these elements and instead suggests a possible synergistic or co-regulatory role in metabolic processes.

Trace elements varied by age, sex, and geographical location, making it difficult to establish baseline values for Cu and Zn. This highlights the need to consider demographic and environmental factors in future research.

These results contribute important baseline data on trace element concentrations in three dolphin species from the Adriatic Sea. The study provides valuable insights into spatial, temporal, and biological factors influencing metal bioaccumulation in marine top predators. These findings have relevance for environmental monitoring and may inform future conservation strategies aimed at protecting cetacean populations in the Adriatic Sea.

## Figures and Tables

**Table 1:** Summary of dolphin specimens stranded along the Croatian Adriatic coast between 1995 and 2013, categorized by species, sex, and age class (where available).

Sample	n	Sex	Age	n (North)	n (South)	Mean Body Length (cm)	Mean Body Weight (cm)
*Tursiops truncatus*	159	81F 77M	52 young 77 adult	68	91	239.2 ± 53.9	150.5 ± 79.8
*Stenella coeruleoalba*	25	10F 15M	-	4	21	191.5 ± 40.3	81.1 ± 15.4
*Grampus griseus*	6	-	6 adult	1	5	300.1 ± 10.6	258.8 ± 46.4

**Table 2:** Recovery percentages of certified reference materials (CRMs), along with the instrumental limits of detection (LOD) and quantification (LOQ) for each analyzed element.

	LOD (mg/kg)	LOQ (mg/kg)	CRM	Certified Value (mg/kg)	Measured Value (mg/kg)	Recovery (%)
Cu	0.01	0.04	DORM-4 ^a^	16.1	15.9 ± 0.9	98.8
			DOLT-5 ^b^	35.4	35.0 ± 2.4	98.9
Zn	0.015	0.051	DORM-4 ^a^	52.4	52.2 ± 3.2	99.6
			DOLT-5 ^b^	105.7	105.3 ± 5.4	99.6

^a^ DORM-4, fish protein (NRC Institute for National Measurement Standards, Otawwa, ON, Canada); ^b^ DOLT-5, dogfish liver (NRC Institute for National Measurement Standards, Otawwa, ON, Canada).

**Table 3:** Mean trace element concentrations (±standard deviation; mg kg^−1^ wet weight) in various tissues of stranded dolphins collected from the Croatian part of the Adriatic Sea.

Species	Tissue (n)	Cu (Mean ± SD)	Zn (Mean ± SD)	Mn (Mean ± SD) [32]
*Tursiops truncatus*	muscle (150)	3.09 ± 4.58	23.68 ± 13.81	0.251 ± 0.220
	kidneys (150)	6.27 ± 3.71	33.13 ± 16.01	0.794 ± 0.354
	liver (147)	18.96 ± 22.24	69.61 ± 44.02	2.87 ± 1.610
	spleen (140)	2.80 ± 2.11	30.80 ± 16.49	0.533 ± 0.340
	lungs (143)	2.88 ± 5.42	31.0 ± 18.79	0.681 ± 3.298
	skin (49)	2.54 ± 2.39	182.4 ± 146.5	0.968 ± 3.297
	adipose tissue (90)	1.47 ± 3.51	17.1 ± 27.6	0.422 ± 1.297
*Stenella coeruleoalba*	muscle (24)	2.49 ± 1.21	13.96 ± 7.11	0.257 ± 0.129
	kidneys (24)	5.43 ± 2.69	36.33 ± 17.42	0.755 ± 0.302
	liver (24)	11.82 ± 7.91	61.68 ± 31.05	3.606 ± 1.903
	spleen (23)	2.08 ± 1.04	28.45 ± 15.93	0.385 ± 0.169
	lungs (23)	1.45 ± 1.00	29.98 ± 16.52	0.307 ± 0.230
	skin (4)	1.99 ± 1.95	233.5 ± 159.02	0.112 ± 0.039
	adipose tissue (13)	0.487 ± 0.252	38.03 ± 43.74	0.104 ± 0.045
*Grampus griseus*	muscle (6)	1.38 ± 0.70	24.97 ± 9.166	0.198 ± 0.058
	kidneys (6)	3.35 ± 0.90	27.84 ± 13.82	1.261 ± 0.564
	liver (6)	6.20 ± 2.22	46.23 ± 13.84	3.998 ± 1.753
	spleen (5)	1.56 ± 0.29	24.48 ± 3.649	0.642 ± 0.279
	lungs (5)	1.43 ± 1.10	11.01 ± 1.643	0.181 ± 0.037
	skin	-	-	-
	adipose tissue	-	-	-

**Table 4:** Reported copper (Cu) concentrations (mg kg^−1^ wet weight) in dolphin species from various regions of the Mediterranean Sea and other geographic regions worldwide.

Species (Number of Individuals)	Muscle (mg/kg)	Kidneys (mg/kg)	Liver (mg/kg)	Spleen (mg/kg)	Lungs (mg/kg)	Skin (mg/kg)	Adipose Tissue (mg/kg)	Location/Period Reference
Mediterranean								
*Tursiops truncatus* (2)	1.18 *	6.55 *	14.61 *	1.23 *	1.41 *			Ligurian sea/1999–2002 [20]
*Tursiops truncatus* (12)	1.19 *	4.26 *	9.61 *		0.93 *			Italian coast/2000–2009 [41]
*Tursiops truncatus* (7)	1.3 *	4.25 *	8 *		2.25 *	0.85 *		Corsica coast/1993–1998 [42]
*Tursiops truncatus* (5)	0.97 *	3.53 *	3.65 *					French coast/1978–1990 [43]
*Tursiops truncatus* (17)	1.2	3.2	8.9			1.1	0.36	Israel/1993–2001 [22]
*Tursiops truncatus* (17)	1.1	3	11.4					Israel/2004–2006 [44]
*Stenella coeruleoalba* (3)	1.2 *	3.26 *	8.69 *	1.03 *	0.73 *			Ligurian sea/1990–2001 [20]
*Stenella coeruleoalba* (3)	1.68 *	3.25 *	7.93 *	1.4 *	0.8 *			Ligurian sea/1986–1990 [45]
*Stenella coeruleoalba* (12–55)	1.57 * median	3.6 * median	5.5 * median			0.63 * median		Tyrrhenian and Ligurian Sea/1987–1994 [46]
*Stenella coeruleoalba* (12)	1.22 *	3.42 *	7.18 *		1.09 *			Italian coast/2000–2009 [41]
*Stenella coeruleoalba* (6)	0.85	1.45	7.73		0.64		0.63	South Italy/1987 [19]
*Stenella coeruleoalba* (10)	1.78	4.06	9.99		1.20		1.21	South Italy/1991 [47]
*Stenella coeruleoalba* (3)	1.95 *	3.25 *	5.25 *		0.675 *	1.125 *		Corsica coast/1993–1998 [42]
*Stenella coeruleoalba* (2)	0.98 *	3.5 *	4.625 *					French coast/1990 [43]
*Stenella coeruleoalba* (55)	1.35 *	2.825 *	8.425 *		0.95 *			Mediterranean Sea France/2002–2009 [48]
*Stenella coeruleoalba* (39)	1.58 *	4.01 *	9.96 *			1.01 *		Mediterranean Sea–Spain/2004–2009 [49]
*Stenella coeruleoalba* (12–55)	1.375 *	4.115 *	8.087 *					Strait of Gibraltar/2012–2013 [50]
*Stenella coeruleoalba* (6)	1.4	2.8	9.7			2.1	0.8	Israel/1993–2001 [22]
*Stenella coeruleoalba* (7)	1.9	4.4	11.8				0.88	Israel coast/2006–2011 [51]
*Grampus griseus* (3)	0.8 *	2.2 *	2.58 *	0.69 *	0.78 *			Ligurian sea/1992–2004 [20]
*Grampus griseus* (2)	1.04 *	3.15 *	2.44 *		0.53 *			Italian coast/2000–2009 [41]
*Grampus griseus* (3)	0.875 *	2.425 *	2.5 *		0.675 *	0.85 *		Corsica coast/1993–1998 [42]
*Grampus griseus* (3)	2.86	2.93	6.11		blood 0.85; stomach content 105	4.73	0.41	Israel/1993–1999 [21]
*Grampus griseus* (1)	0.76	2.81	6.49				0.45	Israel coast/2010 [52]
Other geographic regions worldwide								
*Tursiops truncatus* (2)			7					British Isles/1988–1989 [53]
*Tursiops truncatus* (2)	1.78 *		5 *			1.48 *	0.78 *	Atlantic Ocean–Portugal/1998–2002 [54]
*Tursiops truncatus* (16)		3.78 *	4.35 *			2.13 *	0.78 *	Atlantic Ocean–Portugal/2001–2008 [55]
*Tursiops truncatus* (1)	2.5						1	West Wales/1988 [56]
*Tursiops truncatus* (34)			10.78					South Carolina/1997 [33]
*Tursiops truncatus* (15)			8.9 *			0.81 *		South Carolina/2000–2008 [6]
*Tursiops truncatus* (30)		12 *	17.75 *					Texas/1991–1992 [57]
*Tursiops truncatus* (13)		4 *	6.25 *					Florida/1991–1992 [57]
*Tursiops truncatus* (75)						0.4 *		Florida/2003–2005 [58]
*Tursiops truncatus* (2)			4.570					Brazil/2001–2010 [59]
*Tursiops truncatus* (2)		7.35	13.25					Australia/1995–1996 [10]
*Tursiops truncatus* (11)			21.18					South Australia/1998–2004 [12]
*Tursiops truncatus* (3)			6.91					Hawaiian Islands/1997–2013 [60]
*Tursiops truncatus* (25)	1.626	4.704	11.982					Atlantic Ocean/2005–2013 [61]
*Stenella coeruleoalba* (2)			11					British Isles/1988–1989 [10]
*Stenella coeruleoalba* (1)			5.4					Coast of England and Wales/1994–1996 [10]
*Stenella coeruleoalba* (19)		3.75 *	6.25 *			1.95 *	0.6 *	Atlantic Ocean–Portugal/2001–2008 [55]
*Stenella coeruleoalba* (1)	2.1						0.52	West Wales/1988 [56]
*Stenella coeruleoalba* (1)			8.35 *					Brazilian coast/1997–1999 [5]
*Stenella coeruleoalba* (6)			8.63					Hawaiian Islands/1997–2013 [60]
*Stenella coeruleoalba* (59)	2.04	3.13	8.09					Japan/1977–1980 [62]
*Stenella coeruleoalba* (33)			8.45 *					Japan/1977–1982 [8]

* samples converted from dry weight to wet weight.

**Table 5:** Reported zinc (Zn) concentrations (mg kg^−1^ wet weight) in dolphin species from various regions of the Mediterranean Sea and other geographic regions worldwide.

Species (Number of Individuals)	Muscle (mg/kg)	Kidneys (mg/kg)	Liver (mg/kg)	Spleen (mg/kg)	Lungs (mg/kg)	Skin (mg/kg)	Adipose Tissue (mg/kg)	Location/Period Reference
Mediterranean								
*Tursiops truncatus* (2)	21.5 *	28.25 *	66.38 *	17.5 *	21.75 *			Ligurian sea/1999–2002 [20]
*Tursiops truncatus* (12)	12.363 *	22.54 *	62.41 *		31.39 *			Italian coast/2000–2009 [41]
*Tursiops truncatus* (7)	12.25 *	21 *	30.75 *		24.25 *	116.75 *		Corsica coast/1993–1998 [42]
*Tursiops truncatus* (5)	16.7 *	23.81 *	29.35 *					French coast/1978–1990 [43]
*Tursiops truncatus* (17)	21	18	44			432	10	Israel/1993–2001 [22]
*Tursiops truncatus* (17)	20.8	24.9	49.5					Israel/2004–2006 [44]
*Stenella coeruleoalba* (3)	12.92 *	27.92 *	53.92 *	20.25 *	27.75 *			Ligurian sea/1990–2001 [20]
*Stenella coeruleoalba* (3)	11 *	26 *	34.5 *	24.5 *	30.5 *			Ligurian sea/1986–1990 [45]
*Stenella coeruleoalba* (12–55)	9.365 * median	25.05 * median	27.765 * median			118.8 * median		Tyrrhenian and Ligurian Sea/1987–1994 [46]
*Stenella coeruleoalba* (12)	10.591 *	24.18 *	58.745 *		26.33 *			Italian coast/2000–2009 [41]
*Stenella coeruleoalba* (6)	7.15	15.22	24.65		22.65		5.09	South Italy/1987 [19]
*Stenella coeruleoalba* (10)	12.88	27.71	55.22		28.53		17.03	South Italy/1991 [47]
*Stenella coeruleoalba* (3)	6 *	24.25 *	28 *		28.75 *	94 *		Corsica coast/1993–1998 [42]
*Stenella coeruleoalba* (2)	8.5 *	34.88 *	28.13 *					French coast/1990 [43]
*Stenella coeruleoalba* (55)	9.475 *	23.55 *	35.975 *		26 *			Mediterranean Sea France/2002–2009 [48]
*Stenella coeruleoalba* (39)	16.15 *	25.46 *	46.43 *			169.185 *		Mediterranean Sea–Spain/2004–2009 [49]
*Stenella coeruleoalba* (12–55)	9.4575 *	28.41 *	45.1135 *					Strait of Gibraltar/2012–2013 [50]
*Stenella coeruleoalba* (6)	16	32	48			394	17	Israel/1993–2001 [22]
*Stenella coeruleoalba* (7)	23.2	30.9	69				6.4	Israel coast/2006–2011 [51]
*Grampus griseus* (3)	17.95 *	22.5 *	31 *	13.875 *	11.5 *			Ligurian sea/1992–2004 [20]
*Grampus griseus* (2)	10.253 *	24.604 *	30.54 *		11.26 *			Italian coast/2000–2009 [41]
*Grampus griseus* (3)	22 *	21.75 *	26.75 *		19.25 *	147 *		Corsica coast/1993–1998 [42]
*Grampus griseus* (3)	68.8	32	36.2			1087	12.8	Israel/1993–1999 [21]
*Grampus griseus* (1)	18.2	24.2	53.51				10	Israel coast/2010 [52]
Other geographic regions worldwide								
*Tursiops truncatus* (2)			37					British Isles/1988–1989 [53]
*Tursiops truncatus* (2)	11.3 *		38 *			45 *	5.8 *	Atlantic Ocean–Portugal/1998–2002 [54]
*Tursiops truncatus* (16)		20.9 *	42.8 *			553.3 *	52.5 *	Atlantic Ocean–Portugal/2001–2008 [55]
*Tursiops truncatus* (1)	11						20	West Wales/1988 [56]
*Tursiops truncatus* (34)			56.8					South Carolina/1997 [33]
*Tursiops truncatus* (15)			58 *			213.38 *		South Carolina/2000–2008 [6]
*Tursiops truncatus* (13)		24 *	72.5 *					Texas/1991–1992 [57]
*Tursiops truncatus* (13)		19.5 *	29.5 *					Florida/1991–1992 [57]
*Tursiops truncatus* (74)						171.75 *		Florida/2003–2005 [58]
*Tursiops truncatus* (2)			58.59					Brazil/2001–2010 [59]
*Tursiops truncatus* (2)		33	92.5					Australia/1995–1996 [10]
*Tursiops truncatus* (11)			40.2					South Australia/1998–2004 [12]
*Tursiops truncatus* (3)			70.85					Hawaiian Islands/1997–2013 [60]
*Tursiops truncatus* (25)	12.6	25.987	52.929					Atlantic Ocean/2005–2013 [61]
*Stenella coeruleoalba* (2)			55.5					British Isles/1988–1989 [10]
*Stenella coeruleoalba* (1)			37					Coast of England and Wales/1994–1996 [10]
*Stenella coeruleoalba* (19)		27.03 *	45 *			526 *	35.5 *	Atlantic Ocean–Portugal/2001–2008 [55]
*Stenella coeruleoalba* (1)	11						16.5	West Wales/1988 [56]
*Stenella coeruleoalba* (1)			71.75 *					Brazilian coast/1997–1999 [5]
*Stenella coeruleoalba* (6)			46.56					Hawaiian Islands/1997–2013 [60]
*Stenella coeruleoalba* (59)	11.4	30.1	44.4					Japan/1977–1980 [62]
*Stenella coeruleoalba* (33)			32.25 *					Japan/1977–1982 [8]
*Stenella coeruleoalba* (76)	11.4	30.0	43.7	21.5	20.7	227	5.66	Japan/1977–1980 [75]

* samples converted from dry weight to wet weight.

## Data Availability

The original contributions presented in this study are included in the article. Further inquiries can be directed to the corresponding author.
